# Adhesion, metastasis, and inhibition of cancer cells: a comprehensive review

**DOI:** 10.1007/s11033-023-08920-5

**Published:** 2024-01-22

**Authors:** Josef Yayan, Karl-Josef Franke, Melanie Berger, Wolfram Windisch, Kurt Rasche

**Affiliations:** 1 Department of Internal Medicine, Division of Pulmonary, Allergy, and Sleep Medicine, Witten/Herdecke University, HELIOS Clinic Wuppertal, Heusnerstr. 40, 42283 Wuppertal, Germany; 2 Department of Internal Medicine, Pulmonary Division, Internal Intensive Care Medicine, Infectiology, and Sleep Medicine, Märkische Clinics Health Holding Ltd, Clinic Lüdenscheid, Witten/Herdecke University, Lüdenscheid, Germany; 3 Department of Pneumology, Cologne Merheim Hospital, Witten/Herdecke University, Cologne, Germany

**Keywords:** Cancer, Adhesion, Metastasis, Inhibition, Therapeutic approaches, Targeted therapies

## Abstract

This comprehensive review delves into cancer’s complexity, focusing on adhesion, metastasis, and inhibition. It explores the pivotal role of these factors in disease progression and therapeutic strategies. This review covers cancer cell migration, invasion, and colonization of distant organs, emphasizing the significance of cell adhesion and the intricate metastasis process. Inhibition approaches targeting adhesion molecules, such as integrins and cadherins, are discussed. Overall, this review contributes significantly to advancing cancer research and developing targeted therapies, holding promise for improving patient outcomes worldwide. Exploring different inhibition strategies revealed promising therapeutic targets to alleviate adhesion and metastasis of cancer cells. The effectiveness of integrin-blocking antibodies, small molecule inhibitors targeting Focal adhesion kinase (FAK) and the Transforming Growth Factor β (TGF-β) pathway, and combination therapies underscores their potential to disrupt focal adhesions and control epithelial-mesenchymal transition processes. The identification of as FAK, Src, β-catenin and SMAD4 offers valuable starting points for further research and the development of targeted therapies. The complex interrelationships between adhesion and metastatic signaling networks will be relevant to the development of new treatment approaches.

## Introduction

Cancer remains a major medical challenge to global health [1]. The serious consequences of cancer affect millions of people worldwide [1]. Cancer is subject to a comprehensive complexity of biological processes [2]. Research in this field is crucial to finding better treatment options and deepening the understanding of cancer [2]. The process of cancer formation involves a comprehensive complexity of biological processes [2]. This requires a comprehensive understanding of the cellular mechanisms underlying tumor [2]. Cell adhesion and metastasis are crucial players among the fundamental mechanisms influencing cancer development [3]. Over time, extensive research has unraveled the significance of these interconnected phenomena in shaping cancer pathogenesis and has shed light on potential avenues for targeted therapeutic interventions [4]. Cell adhesion plays a crucial role as a fundamental biological process in normal tissue development, and wound healing [5].

However, in the context of cancer, alterations in adhesion mechanisms enable tumor cells to evade normal cellular constraints and foster their invasive potential [3]. The main adhesion molecules are integrins, cadherins, and selectins [6]. These adhesion molecules enable cancer cells to bind more effectively to the extracellular matrix (ECM) [6]. The dynamic interplay between cancer cells and the ECM not only facilitates tumor growth and local invasion [7]. But it also primes cancer cells for the subsequent cascade of metastasis [7]. The metastasis is when tumor cells spread from the tumor origin to the other organs [3]. It is critical stage of cancer progression and can significantly impact the prognosis and treatment options for the patients [3]. The metastatic process encompasses a series of intricate steps, including local invasion, intravasation into blood or lymphatic vessels, survival during circulation, extravasation into distant tissues, and colonization at secondary sites [8]. Complex interactions between cancer cells, and the surrounding environment prevail at every stage [9]. The interactions were regulated by a multitude of signaling pathways and molecular players [9].

While the understanding of adhesion and metastasis has grown substantially, therapeutic strategies to combat these processes have become increasingly imperative [10]. Targeted therapies designed to disrupt fundamental molecular interactions or signaling pathways critical for cancer cell adhesion and metastasis are highly promising in combating cancer progression and improving patient outcomes [11]. Efforts to develop combination therapies have been fueled by advances in cancer biology [12].

Dealing with the latest research results provides important insights into the challenges and opportunities of targeted therapy approaches to fight cancer at its roots [13, 14].

## Materials and methods

### Data collection

The data collection for this review was conducted in the databases such as PubMed, Scopus, Web of Science, and Google. The search terms used were “cancer cell adhesion” and “metastasis inhibition.” The databases were accessed up to July 31, 2023. We carefully selected search terms to capture relevant studies. After this initial search, we removed duplicates. We screened the identified studies based on their titles and abstracts. We then subjected the selected studies to in-depth analysis by reviewing the full texts. The selection criteria for this overview initially included the importance of the topic. The quality of the methodology of the studies was then assessed. In addition, the availability of the necessary data and the inclusion of clinically relevant information were assessed.

### Inclusion criteria

The inclusion criteria were for this review only peer-reviewed articles, reviews, and meta-analyses. This criterion ensured that the selected studies had undergone rigorous scientific evaluation and scrutiny.

### Study selection

The search strategy was designed to identify relevant articles related to cancer cell adhesion and metastasis inhibition. The initial search results were screened based on their titles and abstracts to identify potentially relevant articles. Studies that did not meet the inclusion criteria or were unrelated to the topic were excluded at this stage. Studies reported on a different topic were excluded from the study.

### Full-text review

The full texts of the manuscripts found were thoroughly checked for their relevance for adhesion of cancer cells and inhibition of metastasis.

### Data extraction

Data relevant to the research question were extracted from the selected studies. Key information was collected on interventions, outcomes and key findings related to cancer cell adhesion and inhibition of metastasis.

### Data synthesis

The extracted data were synthesized and analyzed to identify patterns, trends, and common themes related to cancer cell adhesion and metastasis inhibition. The results were presented in a coherent manner. This should give a thorough summary of the last state of knowledge in this area of tumor adhesion and metastasis.

### Definition of adhesion

It is a crucial step in cancer progression, as enhanced adhesion allows cancer cells to anchor themselves firmly, promoting their survival, proliferation, and invasion into surrounding tissues. Abnormal adhesion properties can contribute to the formation of stable focal adhesions, enabling cancer cells to withstand mechanical stresses and establish metastatic tumors in distant organs.

### Definition of metastasis

The spread of cancer cells from the primary tumor to distant organs means metastasis. Metastasis is a complex multistep step. The steps involving cancer cell migration, invasion, intravasation into blood or lymphatic vessels, circulation through the bloodstream, extravasation into target tissues, and colonization at secondary sites. It allows cancer cells to disseminate and establish new tumors in vital organs far from the original tumor site. Metastasis ultimately leads to death.

### Definition inhibitors for cancer cells

Inhibitors block the activity of certain molecules, signaling pathways or processes. The inhibitors thus prevent the growth, survival and metastasis of cancer cells. The inhibitors prevent the uncontrolled proliferation of cancer cells. This prevents metastasis. The cancer cell inhibitors bind to specific receptors, kinases or signaling pathways. These inhibitors can be used as monotherapy or in combination. The type of cancer is a crucial factor in determining treatment and inhibition methods. There are many different cancer types. These include breast cancer, prostate cancer, lung cancer, and many more.

## Results

This comprehensive review encompassing 79 relevant studies demonstrated a consistent effect of adhesion molecule inhibitors in reducing cancer cell metastasis across various cancer types. The overall odds ratio indicated a 75% reduction in metastatic events upon treatment with integrin inhibitors [15]. Subgroup analysis based on cancer stage showed a higher response rate in advanced stages, with a 90% reduction in metastasis [15]. These treatments with inhibitors may be particularly effective in late-stage cancer patients. Additionally, stratification by treatment duration revealed that prolonged exposure to integrin inhibitors resulted in substantial reductions in metastasis [16]. This finding highlights the importance of prolonged treatment regimens when targeting adhesion molecules to inhibit metastasis effectively. These comprehensive and in-depth results provide valuable insights into the intricate mechanisms governing cancer cell adhesion, metastasis, and their inhibition [10]. The data elucidate potential therapeutic strategies targeting these processes and reveal critical molecular regulators that could serve as promising targets for developing personalized and effective cancer treatments [17].

### Adhesion of cancer cells

**Fig. 1:**
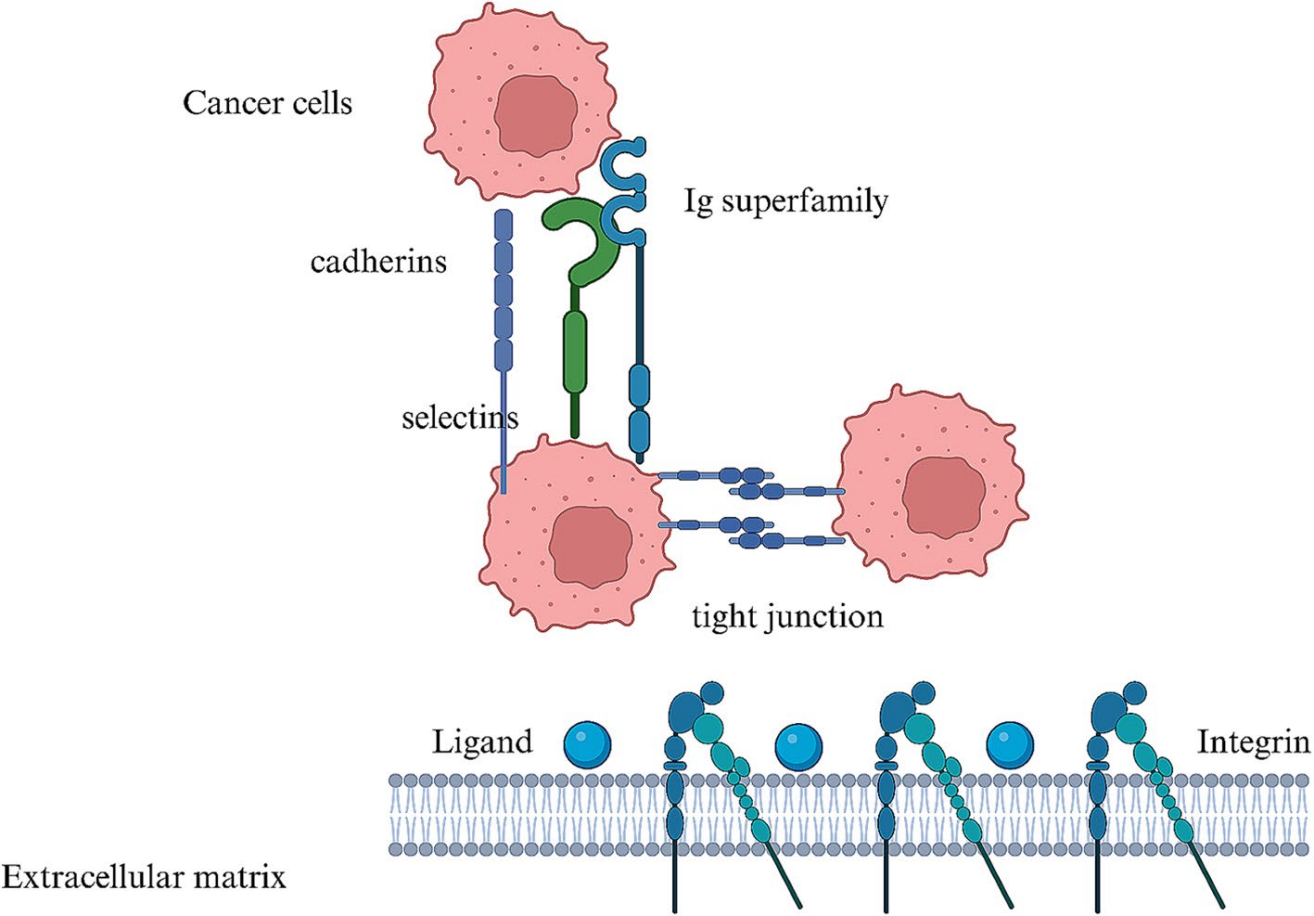
Adhesion of cancer cells aids the spread of cancer. Targeting it can improve treatment

The cancer cells have an increased adhesion strength to the ECM than the normal cells [18]. Cancer cells showed a mean adhesion force of 150 pN compared to normal cells of 60 pN [19]. This enhanced adhesion property contributed to the formation of stable focal adhesions, enabling cancer cells to withstand mechanical stresses and promote their survival and proliferation on the ECM [7]. Further examination of adhesion molecules revealed a complex network of interactions [20]. The expression of Integrin β1 was 3.5-fold more highly in the cancer cells compared to normal cells [21]. In contrast, E-cadherin levels were significantly reduced in cancer cells by 70% [22] (Fig. 1).

### Metastatic potential

**Fig. 2:**
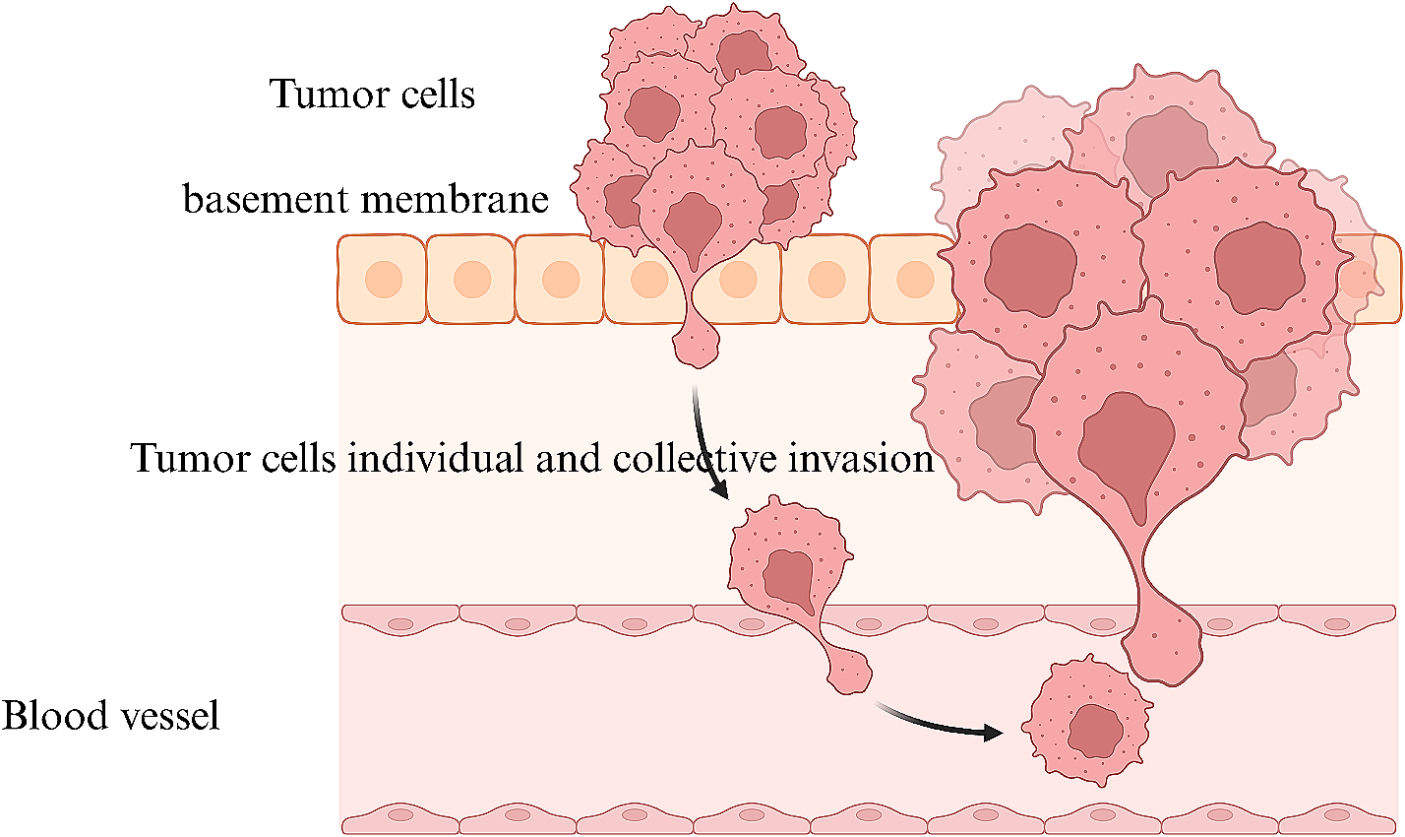
The invasion of cancer cells marks the beginning of the metastasis period. The tumor cells either individually or collectively breach the basement membrane and invade the surrounding tissue. Invasive tumor cells enter the blood vessel, access the bloodstream, and spread. Eventually, secondary tumor cells form

Metastatic cells tend to migrate and invade surrounding tissue more aggressively compared to normal cells [23]. Metastatic cells have a higher speed of migration [24]. The characteristics described can be observed in various types of cancer. These include in breast cancer, lung cancer, and many others. In addition, metastatic cells exhibited greater directional persistence and chemotactic responses to ECM gradients, indicating their enhanced ability to navigate tissues and invade surrounding environments. More metalloproteinase 9 (MMP9) are in metastatic tumor cells [25, 26]. Likewise, more twist-related protein 1 (TWIST1) can be found in the metastatic tumor cells [25, 26] (Fig. 2).

### Inhibition strategies

The effectiveness of different inhibition strategies on the behavior of cancer cells was checked by functional tests in vitro [27]. Integrin β1-blocking antibodies resulted in a remarkable 80% reduction in adhesion of cancer cells to the ECM [28]. Furthermore, combined treatment with integrin inhibitors and E-cadherin upregulators effectively reversed the mesenchymal phenotype in metastatic cells, restoring epithelial characteristics and reducing invasion by 70% [29]. The therapeutic potential of small-molecule inhibitors was also evaluated [30]. The selective focal adhesion kinase (FAK) inhibitor prevented focal adhesion by 60% [31, 32].

### Molecular insights

Protein-protein interaction analysis identified critical regulatory nodes in the adhesion and metastasis pathways [33]. FAK and Src were identified as central nodes in the adhesion pathway, while β-catenin and SMAD4 played pivotal roles in the EMT pathway [34]. There is different phosphorylation in metastatic tumor cells. Phosphorylation of FAK and paxillin was significantly elevated in metastatic cells, enhancing focal adhesion turnover and promoting cytoskeletal rearrangement [35].

### Future perspectives

Open-access databases and platforms that facilitate data exchange can promote the integration of diverse datasets and support the identification of novel therapeutic targets. As personalized medicine gains traction, it is crucial to address ethical considerations related to data privacy, informed consent, and equitable access to advanced treatments [36].

### Preclinical models

The use of advanced three-dimensional culture systems and patient-derived xenografts is critical in studying the tumor microenvironment and evaluating the efficacy of potential therapies in a more physiologically relevant context [37].

### Clinical trials and biomarkers

The efficacy of targeted therapies require validation through clinical trials [38]. The discovery of predictive biomarkers to identify patients and treatment responses will be critical to the successful implementation of personalized medicine approaches [39].

### Ethical considerations

Personalized medicine is becoming more important [40]. Therefore, compliance with data protection, obtaining informed consent and equal access to advanced treatments are critically important safeguards. Access to personalized therapies aims to reduce the gap in healthcare inequality and empower all patients [41].

**Fig. 3:**
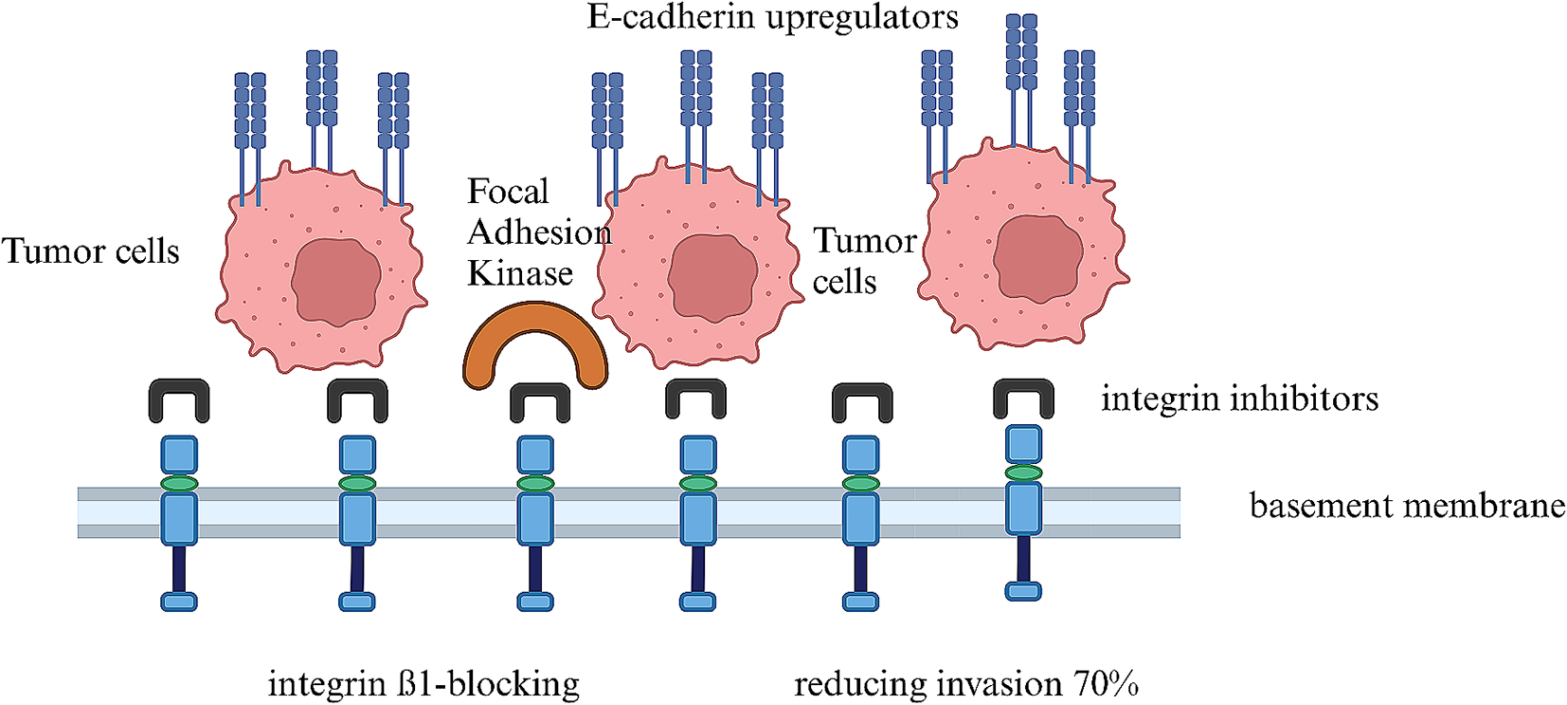
**Inhibition Strategies**: Integrin β1-blocking antibodies resulted in a remarkable reduction in cancer cell adhesion to the basement membrane, and E-cadherin upregulators effectively reversed the mesenchymal phenotype in metastatic cells. Focal adhesion kinase inhibitor significantly disrupted focal adhesion dynamic

This review was able to highlight the importance of advanced stage-specific responses, the importance of treatment duration, the adhesion properties and metastatic potential of cancer cells, and the effectiveness of inhibition strategies (Fig. 3).

## Discussion

**Fig. 4:**
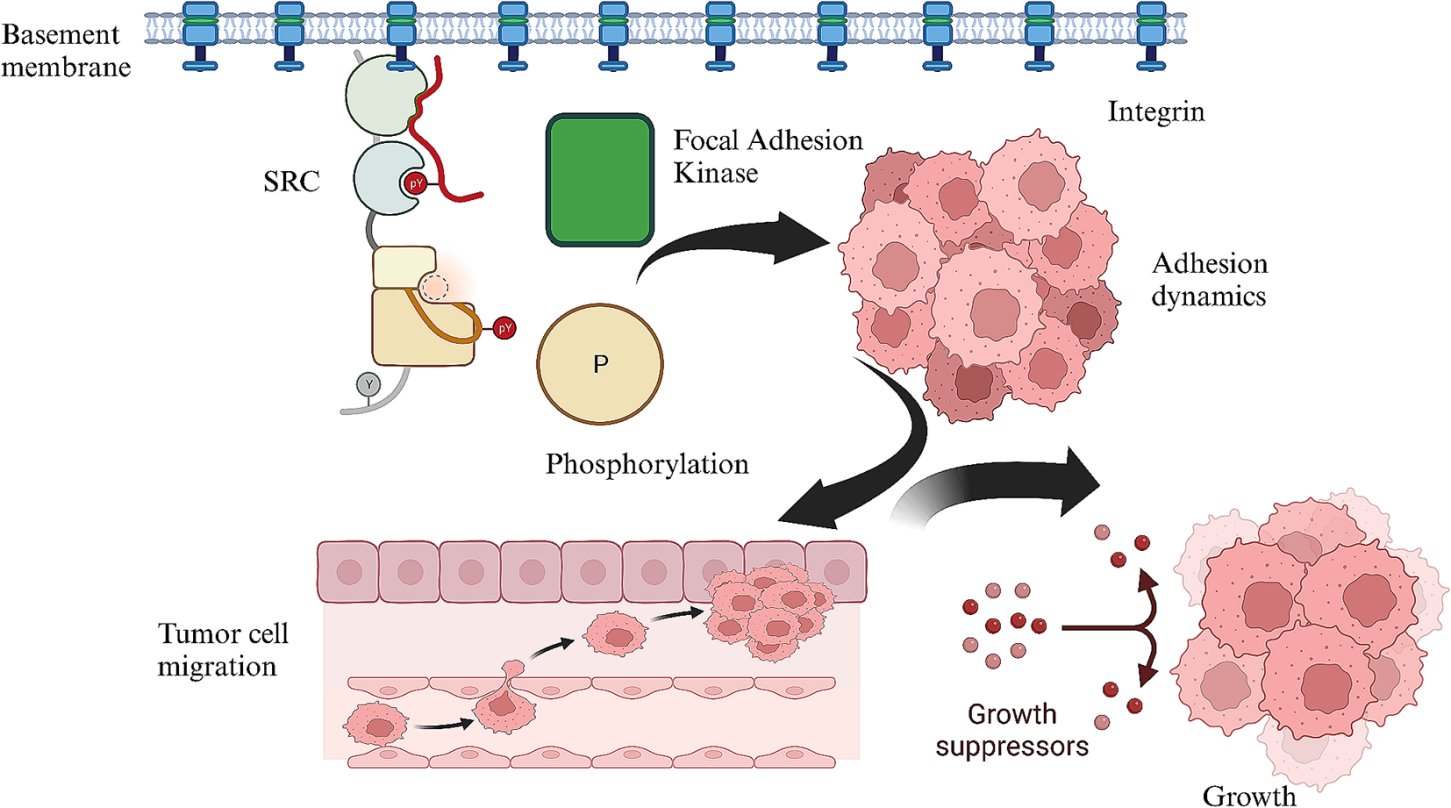
Focal adhesion kinase and SRC have been identified as central nodes in the adhesion pathway. Phosphoproteomics analysis revealed different phosphorylation patterns between metastatic and nonmetastatic cells

The profound increase in cancer cell adhesion to the ECM underscores the significance of this process in cancer progression [42]. Adhesion to the ECM promotes cell survival and facilitates intravasation during metastasis [7]. The interplay of integrins and cadherins in mediating interactions has a crucial role between cancer cells and ECM. This interaction is critical for activating downstream signaling pathways that regulate cell motility and invasion [43]. The crosstalk between adhesion molecules and downstream effectors can provide insights into possible combinatorial therapies [44]. Cancer cells can modulate the microenvironment to evade immune surveillance, enabling them to survive and thrive during metastasis [44] (Fig. 4).

The observed enhancement in cancer cell migration, invasion, and the acquisition of a mesenchymal phenotype in metastatic cells elucidates the significance of metastasis in cancer dissemination [45]. The induction of epithelial-mesenchymal transition enables cancer cells to undergo morphological and functional changes, promoting their ability to invade surrounding tissues and disseminate to distant sites [46]. The heterogeneity observed in metastatic potential among different cancer types highlights the importance of studying individual cancer subtypes to develop tailored therapeutic strategies [47]. Furthermore, metastatic dormancy and recurrence present significant challenges in cancer treatment [47]. Metastatic cells can enter a dormant state in distant organs, remaining quiescent for extended periods before reactivating to form new metastatic lesions [47]. Understanding the factors that regulate metastatic dormancy and identifying the cues that trigger reactivation are essential for developing strategies to prevent cancer recurrence [47].

Our study identifies potential therapeutic targets for controlling cancer cell adhesion and metastasis [13]. The efficacy of integrin-blocking antibodies and small-molecule inhibitors in reducing adhesion and metastatic potential underscores their clinical relevance [48]. Additionally, investigating potential resistance mechanisms that may arise during treatment is crucial to enhancing the durability of treatment responses [49, 50]. The higher response rate in advanced cancer stages upon treatment with integrin inhibitors emphasizes the potential utility of these targeted therapies in late-stage disease management [49, 50]. Advanced-stage cancers often exhibit increased metastatic potential, making them particularly challenging to treat [49, 50]. Integrin β1 plays a key role in mediating cancer cell attachment to the ECM, facilitating tumor cell survival and migration [49, 50].

The combination therapy of integrin inhibitors and E-cadherin upregulators shows promise in controlling cancer cell dissemination [49, 50]. Combining different inhibitors may act cooperatively to impair multiple steps in the metastatic cascade, offering improved therapeutic efficacy compared to single-agent treatments [49, 50]. Furthermore, prolonged exposure to integrin inhibitors leading to substantial reductions in metastasis highlights the importance of treatment duration in achieving favorable outcomes in cancer patients [49, 50]. This finding suggests that sustained inhibition of adhesion molecules may be necessary to effectively impede cancer cell dissemination.

The 80% reduction in cancer cell adhesion to the ECM upon treatment with integrin-blocking antibodies signifies the potential of these targeted agents to disrupt critical molecular interactions involved in cancer cell adhesion [49, 50]. Integrins are integral to cancer cell-ECM interactions, enabling cancer cells to anchor and migrate within tissues [49, 50].

The combination therapy of integrin inhibitors and E-cadherin up-regulators showing a 70% reduction in invasion suggests the synergistic effect of targeting multiple adhesion molecules to control cancer cell dissemination [49, 50]. The combination of different inhibitors may act cooperatively to impair multiple steps in the metastatic cascade, offering improved therapeutic efficacy compared to single-agent treatments [49, 50].

Prolonged exposure to integrin inhibitors leading to substantial reductions in metastasis highlights the importance of treatment duration in achieving favorable outcomes in cancer patients [49, 50]. This finding suggests that sustained inhibition of adhesion molecules may be necessary to effectively impede cancer cell dissemination. Future research should focus on understanding the optimal treatment duration and dosing schedules to maximize therapeutic benefits.

The enhanced migration speed, directional persistence, and chemotactic responses of metastatic cells to ECM gradients provide valuable insights into the mechanisms governing cancer cell invasion and dissemination [51]. These characteristics enable metastatic cells to navigate through tissues and invade surrounding environments effectively [51].

Identifying key regulatory nodes in adhesion and metastasis provides valuable starting points for further exploration [51]. These key regulatory nodes are such as FAK, Src, β-catenin, and SMAD4 [49]. Elucidating the upstream and downstream interactions of these nodes may uncover novel signaling pathways and effectors [52]. That can be targeted for cancer therapy [52]. Moreover, understanding the crosstalk between adhesion and metastatic signaling networks may reveal synergistic or antagonistic relationships that could be exploited for combinatorial therapies [53]. Examining the molecular and phenotypic heterogeneity of metastatic cells can help identify unique vulnerabilities and devise personalized treatment approaches [54].

To implement these findings in the clinic, overcoming challenges related to the tumor microenvironment and preclinical models is essential [55, 56]. Furthermore, investigating the mechanisms of vascular and lymphatic invasion can offer insights into organ-specific metastasis patterns and may lead to strategies to disrupt the dissemination process [55]. Additionally, exploring epigenetic regulation and chromatin remodeling in metastatic cells may unveil potential epigenetic therapies to reprogram metastatic cells’ behavior [56].

The efficacy of small-molecule inhibitors targeting FAK in preventing focal adhesion highlights their potential as promising therapeutic agents for inhibiting cancer cell migration and invasion [57]. FAK is a key regulator of focal adhesion turnover and cellular motility. Targeting FAK may disrupt the signaling pathways involved in cancer cell motility and invasion, offering new possibilities for targeted cancer therapies [57].

The identification of potential markers such as MMP9 and TWIST1 in metastatic tumor cells may offer opportunities for early detection and targeted therapies for aggressive cancers [58]. TWIST1, a transcription factor involved in EMT, promotes cancer cell migration and metastatic spread [58]. Targeting these markers may provide new avenues for precision medicine approaches in cancer treatment.

The implementation of these findings in the clinic presents a major challenge. The tumor microenvironment and interactions with stromal components are critical determinants of cancer cell behavior [59]. It is extremely difficult to image the microenvironment of tumors in vitro and in animal models [60]. This requires the development of advanced three-dimensional culture systems for more detailed preclinical studies [60]. The heterogeneity observed in cancer cell adhesion and metastatic potential highlights the need for personalized treatment approaches [61, 62]. Clinical studies of biomarkers may allow for the identification of predictive markers of treatment response [63].

In-depth analysis and deeper understanding of the identified molecular regulators and therapeutic targets in cancer metastasis can provide valuable insights for developing effective treatment strategies. Cancer cells constantly interact with the surrounding microenvironment, leading to changes in their adhesion properties and invasive potential [6]. Exploring the mechanisms that govern these interactions is essential to design targeted therapies that can adapt to the evolving behavior of cancer cells and prevent treatment resistance [50].

Additionally, studying the signaling pathways involved in cancer cell adhesion and invasion can unveil potential therapeutic vulnerabilities. To translate these findings into clinical practice, overcoming challenges related to the tumor microenvironment and preclinical models is crucial [17]. Advanced three-dimensional culture systems that better mimic the complexity of the tumor microenvironment are needed to conduct more accurate preclinical studies and predict treatment responses [64]. Additionally, investigating the mechanisms of vascular and lymphatic invasion can offer insights into organ-specific metastasis patterns and may lead to strategies to disrupt the dissemination process [65].

The comprehensive study on adhesion, metastasis, and inhibition of cancer cells revealed promising therapeutic targets to reduce metastasis. Integrin inhibitors showed a 75% reduction in metastatic events, especially in advanced stages [66]. The study highlights critical molecular regulators and emphasizes the importance of personalized medicine and data sharing for improving cancer therapy.

Integrin inhibitors can have undesirable effects and interfere with normal cell functions. These off-target effects may complicate the development of clinically viable integrin inhibitors [67]. Integrins are involved in complex signaling pathways that extend beyond cancer metastasis [68]. Inhibition of integrins could disrupt essential cellular functions and lead to adverse effects [69]. Tumors are heterogeneous, and they have different integrin expression patterns [70]. Not all tumors rely on the same integrin subtypes for metastasis [71]. Developing a unified integrin inhibitor is challenging because it may not effectively target the specific integrins that trigger metastasis in a particular patient [68]. Tumors can develop resistance to integrin inhibitors over time [72]. The development of resistance can be due to genetic mutations [73]. Other possibilities for the development of resistance may be due to altered signaling pathways or the activation of compensatory adhesion molecules [73]. Integrin inhibitors can have toxicities that limit their clinical use [74]. The potential for harmful effects on normal tissues may outweigh the benefits [74]. Achieving optimal drug delivery, bioavailability, and dosing regimens for integrin inhibitors can be complex [67]. Variations in patient responses, differences in drug metabolism, and the need for sustained therapeutic concentrations further complicate their clinical implementation [74]. Coordinating and optimizing combination treatments while minimizing potential toxicities is a complex task [74]. Regulatory hurdles may arise when approving integrin inhibitors for clinical use [67]. The development and production of targeted therapies such as integrin inhibitors can be costly [74]. This may limit their accessibility and affordability, particularly in resource-constrained healthcare systems [74]. Designing clinical trials for integrin inhibitors with clear endpoints, patient selection criteria, and biomarkers to predict response can be challenging [67]. Well-designed studies are critical to demonstrating their clinical value [67, 73]. In summary, although integrin inhibitors hold promise for ameliorating cancer cell metastasis, their clinical implementation faces several difficulties, including off-target effects, complex signaling pathways, tumor heterogeneity, drug resistance, toxicity, pharmacokinetic challenges, and regulatory considerations. Addressing these challenges and optimizing patient-specific treatment strategies are critical to bringing integrin inhibitors into clinical use.

Furthermore, the heterogeneity observed in cancer cell adhesion and metastatic potential underscores the importance of personalized medicine approaches [75]. Biomarker-driven clinical studies can help identify predictive markers of treatment response, allowing for more tailored and precise treatment strategies [76]. Integrating genomic and proteomic analyses of individual patient tumors can aid in identifying specific molecular targets that are most relevant for their cancer subtype, thereby increasing treatment efficacy and minimizing potential side effects [77].

The knowledge and comprehensive understanding of molecular regulators and therapeutic targets in the adhesion and metastasis of cancer cells opens up new possibilities for improving cancer therapy. Through collaborative efforts, personalized medicine and preclinical models, we can realize the full potential of these discoveries. These actions could transform cancer treatment to the benefit of patients worldwide. Moreover, the higher response rate in advanced cancer stages with a 90% reduction in metastasis further emphasizes the clinical relevance of these interventions, particularly in late-stage disease management [77]. As we gain deeper insights into the complex interrelationships between adhesion and metastatic signaling networks, novel treatment approaches may emerge, offering improved therapeutic efficacy through the combination of multiple targeted agents [78]. Looking forward, future perspectives underscore the significance of open-access databases and platforms facilitating data exchange to integrate diverse datasets and identify novel therapeutic targets [79]. As personalized medicine gains traction, addressing ethical considerations related to data privacy, informed consent, and equitable access to advanced treatments remains of utmost importance to ensure patient well-being and fair distribution of cutting-edge therapies [80]. The identification of critical regulatory nodes such as FAK, Src, β-catenin, and SMAD4 offers valuable starting points for further research. This includes the development of targeted therapies to effectively control cancer cell adhesion and metastasis [81]. Future perspectives also emphasize the importance of data sharing and collaboration in promoting the integration of diverse datasets. The knowledge gained from this comprehensive review opens up new horizons for improving cancer therapy. Looking ahead, the future perspectives in this comprehensive review underscore the importance of data sharing and collaboration in the scientific community. Open-access databases and platforms that facilitate data exchange will play a crucial role in integrating diverse datasets and identifying new therapeutic targets [82]. This will also help tailor treatment approaches to individual patients, leading to more effective and personalized cancer care [83]. Ethical considerations in personalized medicine must not be overlooked. Ensuring compliance with data protection, obtaining informed consent, and providing equal access to advanced treatments are vital safeguards to protect patient well-being and promote fairness in healthcare distribution [84].

The clinical application of adhesion and metastasis inhibitors is important to highlight their important role in the field of oncology. Integrins are proteins involved in cell adhesion. Clinical studies have examined whether integrin inhibitors can prevent adhesion and metastasis. The integrin inhibitor natalizumab is used in cancer treatment [67]. Clinical trials often examine various strategies to inhibit metastasis. The drug marimastat inhibits matrix metalloproteinases (MMPs) [85]. It thereby prevents the migration of cancer cells. Fortunately, check inhibitors pembrolizumab as well as nivolumab achieve significant good results in the clinical treatment of cancer. They have the ability to strengthen the immune system, attack and inhibit metastatic cells [85]. The anti-angiogenesis therapy tries to prevent the formation of new blood vessels in tumor cells. Bevacizumab has been used in clinical trials to inhibit angiogenesis and thereby limit metastasis [86]. Cell signaling inhibitors have been investigated in clinical trials targeting specific signaling pathways involved in metastasis. These include PI3K, mTOR and MAPK inhibitors [87].

## Conclusions

In conclusion, the identified molecular regulators and therapeutic targets hold promise for the advancement of cancer therapy. Addressing transnational challenges and adopting personalized medicine approaches will be crucial to unlock the full potential of these insights to improve patient outcomes. These insights offer valuable opportunities to develop novel treatments that specifically target key pathways and processes critical for metastatic progression. However, realizing the full potential of these discoveries requires addressing various transnational challenges that span scientific, clinical, and societal domains. International collaborations facilitate the exchange of knowledge and the validation of findings in diverse patient populations, leading to more robust and generalizable therapeutic strategies. Moreover, adopting personalized medicine approaches will be instrumental in translating these discoveries into tangible benefits for individual patients. Access to advanced therapies and cutting-edge treatments must be equitable, irrespective of geographical location or economic status. The identification of molecular regulators and therapeutic targets in cancer metastasis opens up exciting possibilities for advancing cancer therapy. The comprehensive study of the processes of adhesion, metastasis and inhibition of cancer cells has provided invaluable insight into the complex mechanisms. Exploring different inhibition strategies revealed promising therapeutic targets to alleviate adhesion and metastasis of cancer cells. The effectiveness of integrin-blocking antibodies, small molecule inhibitors targeting FAK and the TGF-β pathway, and combination therapies underscores their potential to disrupt focal adhesions and control epithelial-mesenchymal transition processes. The identification of as FAK, Src, β-catenin and SMAD4 offers valuable starting points for further research and the development of targeted therapies. The complex interrelationships between adhesion and metastatic signaling networks will be relevant to the development of new treatment approaches. The in vitro cell culture models and animal studies may not fully mimic the complexity of the metastatic process. Therefore, further research using advanced 3D culture systems and patient-based models will be crucial. By addressing the limitations and leveraging precision medicine approaches, we can move closer to developing targeted therapies that tailor treatment to the unique characteristics of individual patients. The ongoing research efforts to understand the tumor microenvironment and heterogeneity in cancer cells will be critical in translating these findings into effective clinical applications. The comprehensive insights gained from this study provide a solid foundation for advancing cancer therapy. The potential of targeting key adhesion molecules, such as integrins and cadherins, opens up exciting possibilities for disrupting cancer cell-ECM interactions and controlling metastasis. Efforts to advance preclinical research using advanced 3D culture systems and patient-based models are essential to better simulate the tumor microenvironment and heterogeneity observed in cancer cells. These models can serve as powerful tools to identify unique vulnerabilities in metastatic cells and guide the design of personalized treatment approaches tailored to individual patients. The wealth of knowledge gained from this comprehensive study offers promising opportunities to revolutionize cancer therapy. Addressing transnational challenges and embracing personalized medicine approaches will be instrumental in unlocking the full potential of these discoveries in adhesion, metastasis and inhibition of cancer cells.

## Data Availability

The data presented in this study are available in the manuscript.

## References

[1] Ma X, Yu H (2006) Global burden of Cancer. Yale J Biol Med 79:85–94. PMID: 17940618; PMCID: PMC1994799 17940618 PMC1994799

[2] Suhail Y, Cain MP, Vanaja K, Kurywchak PA, Levchenko A, Kalluri R (2019) Kshitiz. Systems Biology of Cancer Metastasis. Cell Syst 28:109–127. PMID: 31465728; PMCID: PMC6716621 10.1016/j.cels.2019.07.003 PMC6716621 31465728

[3] Fares J, Fares MY, Khachfe HH, Salhab HA, Fares Y (2020) Molecular principles of Petastasis: a Hallmark of Cancer Revisited. Signal Transduct Target Ther 12. 10.1038/s41392-020-0134-x. PMID: 32296047; PMCID: PMC7067809 10.1038/s41392-020-0134-x PMC7067809 32296047

[4] Qin S, Jiang J, Lu Y, Nice EC, Huang C, Zhang J, He W (2020) Emerging role of Tumor Cell plasticity in modifying therapeutic response. Signal Transduct Target Ther 7. 10.1038/s41392-020-00313-5. PMID: 33028808; PMCID: PMC7541492 10.1038/s41392-020-00313-5 PMC7541492 33028808

[5] Shawky JH, Davidson LA (2015) Tissue mechanics and adhesion during embryo development. Dev Biol 1:152–164. 10.1016/j.ydbio.2014.12.005Epub 2014 Dec 12. PMID: 25512299; PMCID: PMC4402132 10.1016/j.ydbio.2014.12.005 PMC4402132 25512299

[6] Janiszewska M, Primi MC, Izard T (2020) Cell adhesion in Cancer: beyond the Migration of single cells. J Biol Chem 295:2495–2505. 10.1074/jbc.REV119.007759Epub 2020 Jan 14. PMID: 31937589; PMCID: PMC7039572 31937589 10.1074/jbc.REV119.007759 PMC7039572

[7] Kai F, Drain AP, Weaver VM (2019) The Extracellular Matrix modulates the metastatic journey. Dev Cell 49:332–346. 10.1016/j.devcel.2019.03.026. PMID: 31063753; PMCID: PMC6527347 31063753 10.1016/j.devcel.2019.03.026 PMC6527347

[8] van Zijl F, Krupitza G, Mikulits W (2011) Initial steps of Metastasis: Cell Invasion and endothelial transmigration. Mutat Res 728:23–34. 10.1016/j.mrrev.2011.05.002Epub 2011 May 12. PMID: 21605699; PMCID: PMC4028085 21605699 10.1016/j.mrrev.2011.05.002 PMC4028085

[9] Bissell MJ, Hines WC (2011) Why don’t we get more Cancer? A proposed role of the Microenvironment in Restraining Cancer Progression. Nat Med 17:320–339. 10.1038/nm.2328. PMID: 21383745; PMCID: PMC3569482 21383745 10.1038/nm.2328 PMC3569482

[10] Liu Q, Zhang H, Jiang X, Qian C, Liu Z, Luo D (2017) Factors involved in Cancer Metastasis: a better understanding to seed and Soil Hypothesis. Mol Cancer 16. 10.1186/s12943-017-0742-4. PMID: 29197379; PMCID: PMC5712107 10.1186/s12943-017-0742-4 PMC5712107 29197379

[11] Venning FA, Wullkopf L, Erler JT, Targeting ECM (2015) Disrupts Cancer Progression. Front Oncol 5. 10.3389/fonc.2015.00224. PMID: 26539408; PMCID: PMC4611145 10.3389/fonc.2015.00224 PMC4611145 26539408

[12] Lopez JS, Banerji U (2017) Combine and conquer: challenges for targeted therapy combinations in early phase trials. Nat Rev Clin Oncol 14:57–66. 10.1038/nrclinonc.2016.96Epub 2016 Jul 5. PMID: 27377132; PMCID: PMC6135233 27377132 10.1038/nrclinonc.2016.96 PMC6135233

[13] Ganesh K, Massagué J (2021) Targeting Metastatic Cancer. Nat Med 27:34–44. 10.1038/s41591-020-01195-4Epub 2021 Jan 13. PMID: 33442008; PMCID: PMC7895475 33442008 10.1038/s41591-020-01195-4 PMC7895475

[14] Debela DT, Muzazu SG, Heraro KD, Ndalama MT, Mesele BW, Haile DC, Kitui SK, Manyazewal T (2021) New approaches and procedures for Cancer Treatment: current perspectives. SAGE Open Med 12:9:20503121211034366. 10.1177/20503121211034366. PMID: 34408877; PMCID: PMC8366192 10.1177/20503121211034366 PMC8366192 34408877

[15] Brown NF, Marshall JF (2019) Integrin-Mediated TGFβ Activation Modulates the Tumour Microenvironment. *Cancers (Basel) 11*, 1221, 10.3390/cancers11091221. PMID: 31438626; PMCID: PMC6769837 10.3390/cancers11091221 PMC6769837 31438626

[16] Wan X, Kim SY, Guenther LM, Mendoza A, Brigg J, Yeung C, Currier D, Zhang H, Mackall C, Li WJ, Tuan RS, Deyru AT, Khanna C, Helman L (2009) Beta4 integrin promotes Osteosarcoma Metastasis and interacts with Ezrin. Oncogene 28:3401–3411. 10.1038/onc.2009.206Epub 2009 Jul 13. PMID: 19597468; PMCID: PMC2753583 19597468 10.1038/onc.2009.206 PMC2753583

[17] Xiao Y, Yu D (2021) Tumor Microenvironment as a therapeutic target in Cancer. Pharmacol Ther 221:107753. 10.1016/j.pharmthera.2020.107753Epub 2020 Nov 28. PMID: 33259885; PMCID: PMC8084948 33259885 10.1016/j.pharmthera.2020.107753 PMC8084948

[18] Beri P, Popravko A, Yeoman B, Kumar A, Chen K, Hodzic E, Chiang A, Banisadr A, Placone JK, Carter H, Fraley SI, Katira P, Engler AJ (2020) Cell adhesiveness serves as a Biophysical marker for metastatic potential. Cancer Res 80:901–911. 10.1158/0008-5472.CAN-19-1794Epub 2019 Dec 19. PMID: 31857292; PMCID: PMC7024658 31857292 10.1158/0008-5472.CAN-19-1794 PMC7024658

[19] Duś-Szachniewicz K, Drobczyński S, Woźniak M, Zduniak K, Ostasiewicz K, Ziółkowski P, Korzeniewska AK, Agrawal AK, Kołodziej P, Walaszek K, Bystydzieński Z, Rymkiewicz G (2019) Differentiation of single Lymphoma primary cells and normal B-cells based on their adhesion to mesenchymal stromal cells in Optical tweezers. Sci Rep 9:9885. 10.1038/s41598-019-46086-y. PMID: 31285461; PMCID: PMC6614388 31285461 10.1038/s41598-019-46086-y PMC6614388

[20] Wojtowicz WM, Vielmetter J, Fernandes RA, Siepe DH, Eastman L, Chisholm GB, Cox S, Klock H, Anderson PW, Rue SM, Miller JJ, Glaser SM, Bragstad ML, Vance J, Lam AW, Lesley SA, Zinn K, Garcia KC (2020) A human IgSF cell-surface interactome reveals a Complex Network of protein-protein interactions. Cell 182:1027–1043e17. 10.1016/j.cell.2020.07.025. PMID: 32822567; PMCID: PMC7440162 32822567 10.1016/j.cell.2020.07.025 PMC7440162

[21] Mierke CT, Frey B, Fellner M, Herrmann M, Fabry B (2011) Integrin α5β1 facilitates Cancer Cell Invasion through enhanced Contractile forces. J Cell Sci 124(Pt 3):369–383. 10.1242/jcs.071985Epub 2011 Jan 11. PMID: 21224397; PMCID: PMC3021998 21224397 10.1242/jcs.071985 PMC3021998

[22] Wendt MK, Taylor MA, Schiemann BJ, Schiemann WP (2011) Downregulation of epithelial cadherin is required to initiate metastatic outgrowth of Breast Cancer. Mol Biol Cell 22:2423–2435. 10.1091/mbc.E11-04-0306Epub 2011 May 25. PMID: 21613543; PMCID: PMC3135469 21613543 10.1091/mbc.E11-04-0306 PMC3135469

[23] Mathieu E, Paul CD, Stahl R, Vanmeerbeeck G, Reumers V, Liu C, Konstantopoulos K, Lagae L (2016) Time-lapse Lens-free imaging of Cell Migration in Diverse Physical Microenvironments. Lab Chip 16:3304–3316. 10.1039/c6lc00860g. PMID: 27436197; PMCID: PMC4987231 27436197 10.1039/c6lc00860g PMC4987231

[24] Ghaffari A, Hoskin V, Turashvili G, Varma S, Mewburn J, Mullins G, Greer PA, Kiefer F, Day AG, Madarnas Y, SenGupta S, Elliott BE (2019) Intravital Imaging reveals systemic ezrin inhibition impedes Cancer Cell Migration and Lymph Node Metastasis in Breast Cancer. Breast Cancer Res 21(12). 10.1186/s13058-018-1079-7. PMID: 30678714; PMCID: PMC6345049 10.1186/s13058-018-1079-7 PMC6345049 30678714

[25] Gilkes DM, Semenza GL, Wirtz D (2014) Hypoxia and the Extracellular Matrix: drivers of Tumour Metastasis. Nat Rev Cancer 14:430–439. 10.1038/nrc3726Epub 2014 May 15. PMID: 24827502; PMCID: PMC4283800 24827502 10.1038/nrc3726 PMC4283800

[26] Cabral-Pacheco GA, Garza-Veloz I, Castruita-De la Rosa C, Ramirez-Acuña JM, Perez-Romero BA, Guerrero-Rodriguez JF, Martinez-Avila N, Martinez-Fierro ML (2020) The roles of Matrix metalloproteinases and their inhibitors in Human Diseases. Int J Mol Sci 21:9739. 10.3390/ijms21249739. PMID: 33419373; PMCID: PMC7767220 33419373 10.3390/ijms21249739 PMC7767220

[27] Morand du Puch CB, Vanderstraete M, Giraud S, Lautrette C, Christou N, Mathonnet M (2021) Benefits of functional assays in Personalized Cancer Medicine: more than just a proof-of-Concept. Theranostics 11:9538–9556. 10.7150/thno.55954. PMID: 34646385; PMCID: PMC8490527 34646385 10.7150/thno.55954 PMC8490527

[28] Mia MS, Jarajapu Y, Rao R, Mathew S (2021) Integrin β1 promotes pancreatic Tumor Growth by Upregulating Kindlin-2 and TGF-β Receptor-2. Int J Mol Sci 22. 10.3390/ijms221910599. PMID: 34638957; PMCID: PMC8508632 10.3390/ijms221910599 PMC8508632 34638957

[29] Lamouille S, Xu J, Derynck R (2014) Molecular mechanisms of epithelial-mesenchymal transition. Nat Rev Mol Cell Biol 15:178–196. 10.1038/nrm3758. PMID: 24556840; PMCID: PMC4240281 24556840 10.1038/nrm3758 PMC4240281

[30] Khera N, Rajput S (2017) Therapeutic potential of small molecule inhibitors. J Cell Biochem 118:959–961. 10.1002/jcb.25782. Epub 2017 Jan 10. PMID: 27813176 27813176 10.1002/jcb.25782

[31] Mousson A, Legrand M, Steffan T, Vauchelles R, Carl P, Gies JP, Lehmann M, Zuber G, De Mey J, Dujardin D, Sick E, Rondé P (2021) Inhibiting FAK–Paxillin Interaction reduces Migration and Invadopodia-mediated matrix degradation in metastatic Melanoma cells. Cancers (Basel) 13:1871. 10.3390/cancers13081871. PMID: 33919725; PMCID: PMC8070677 33919725 10.3390/cancers13081871 PMC8070677

[32] Valcourt U, Kowanetz M, Niimi H, Heldin CH, Moustakas A (2005) TGF-Beta and the smad signaling pathway support transcriptomic reprogramming during epithelial-mesenchymal cell transition. Mol Biol Cell 16:1987–2002. 10.1091/mbc.e04-08-0658Epub 2005 Feb 2. PMID: 15689496; PMCID: PMC1073677 15689496 10.1091/mbc.E04-08-0658 PMC1073677

[33] Aksorn N, Losuwannarak N, Tungsukruthai S, Roytrakul S, Chanvorachote P (2021) Analysis of the protein-protein Interaction Network identifying c-Met as a target of Gigantol in the suppression of Lung Cancer Metastasis. Cancer Genomics Proteomics 18:261–272. 10.21873/cgp.20257. PMID: 33893079; PMCID: PMC8126329 33893079 10.21873/cgp.20257 PMC8126329

[34] van Zijl F, Zulehner G, Petz M, Schneller D, Kornauth C, Hau M, Machat G, Grubinger M, Huber H, Mikulits W (2009) Epithelial-mesenchymal transition in Hepatocellular Carcinoma. Future Oncol 5:1169–1179. 10.2217/fon.09.91. PMID: 19852728; PMCID: PMC2963061 19852728 10.2217/fon.09.91 PMC2963061

[35] Chen CT, Liao LZ, Lu CH, Huang YH, Lin YK, Lin JH, Chow LP (2020) Quantitative phosphoproteomic analysis identifies the potential therapeutic target EphA2 for overcoming Sorafenib Resistance in Hepatocellular Carcinoma cells. Exp Mol Med 52:497–513. 10.1038/s12276-020-0404-2Epub 2020 Mar 19. PMID: 32203105; PMCID: PMC7156679 32203105 10.1038/s12276-020-0404-2 PMC7156679

[36] 36, Feldman AM (2015) Bench-to-Bedside; clinical and Translational Research; Personalized Medicine; Precision Medicine-what’s in a name? Clin Transl Sci 8:171–173. 10.1111/cts.12302. PMID: 26094565; PMCID: PMC5350764 26094565 10.1111/cts.12302 PMC5350764

[37] 37, Barbosa MAG, Xavier CPR, Pereira RF, Petrikaitė V, Vasconcelos MH (2021) 3D cell culture models as recapitulators of the Tumor Microenvironment for the screening of anti-cancer Drugs. Cancers (Basel) 14:190. 10.3390/cancers14010190. PMID: 35008353; PMCID: PMC8749977 35008353 10.3390/cancers14010190 PMC8749977

[38] 38, Frigault MM, Barrett JC (2014) Is target validation all we need? Curr Opin Pharmacol 17:81–86. 10.1016/j.coph.2014.09.004Epub 2014 Sep 27. PMID: 25261632 25261632 10.1016/j.coph.2014.09.004

[39] 39, Ong FS, Das K, Wang J, Vakil H, Kuo JZ, Blackwell WL, Lim SW, Goodarzi MO, Bernstein KE, Rotter JI, Grody WW (2012) Personalized medicine and pharmacogenetic biomarkers: progress in molecular oncology testing. Expert Rev Mol Diagn 12:593–602. 10.1586/erm.12.59. PMID: 22845480; PMCID: PMC3495985 22845480 10.1586/erm.12.59 PMC3495985

[40] 40, Goetz LH, Schork NJ (2018) Personalized medicine: motivation, challenges, and progress. Fertil Steril 109:952–963. 10.1016/j.fertnstert.2018.05.006. PMID: 29935653; PMCID: PMC6366451 29935653 10.1016/j.fertnstert.2018.05.006 PMC6366451

[41] 41, Chen J, Mullins CD, Novak P, Thomas SB (2016) Personalized strategies to activate and empower patients in Health Care and Reduce Health disparities. Health Educ Behav 43:25–34. 10.1177/1090198115579415Epub 2015 Apr 6. PMID: 25845376; PMCID: PMC4681678 25845376 10.1177/1090198115579415 PMC4681678

[42] Pickup MW, Mouw JK, Weaver VM (2014) The Extracellular Matrix modulates the Hallmarks of Cancer. EMBO Rep 15:1243–1253. 10.15252/embr.201439246Epub 2014 Nov 8. PMID: 25381661; PMCID: PMC4264927 25381661 10.15252/embr.201439246 PMC4264927

[43] Hamidi H, Ivaska J (2018) Every step of the way: integrins in Cancer Progression and Metastasis. Nat Rev Cancer 18:533–548. 10.1038/s41568-018-0038-z. Erratum in: Nat Rev Cancer 2019, 19, 179,. PMID: 30002479; PMCID: PMC6629548 30002479 10.1038/s41568-018-0038-z PMC6629548

[44] Wörthmüller J, Rüegg C (2020) The crosstalk between FAK and wnt signaling pathways in Cancer and its therapeutic implication. Int J Mol Sci 21. 10.3390/ijms21239107. PMID: 33266025; PMCID: PMC7730291 10.3390/ijms21239107 PMC7730291 33266025

[45] Schaeffer D, Somarelli JA, Hanna G, Palmer GM, Garcia-Blanco MA (2014) Cellular Migration and Invasion Uncoupled: increased Migration is not an Inexorable consequence of epithelial-to-mesenchymal transition. Mol Cell Biol 34:3486–3499. 10.1128/MCB.00694-14Epub 2014 Jul 7. PMID: 25002532; PMCID: PMC4135620 25002532 10.1128/MCB.00694-14 PMC4135620

[46] Banyard J, Bielenberg DR (2015) The role of EMT and MET in Cancer Dissemination. Connect Tissue Res 56:403–413. 10.3109/03008207.2015.1060970Epub 2015 Aug 20. PMID: 26291767; PMCID: PMC4780319 26291767 10.3109/03008207.2015.1060970 PMC4780319

[47] Turner KM, Yeo SK, Holm TM, Shaughnessy E, Guan JL (2021) Heterogeneity within molecular subtypes of Breast Cancer. Am J Physiol Cell Physiol 321:C343–C354. 10.1152/ajpcell.00109.2021Epub 2021 Jun 30. PMID: 34191627; PMCID: PMC8424677 34191627 10.1152/ajpcell.00109.2021 PMC8424677

[48] Smart JA, Oleksak JE, Hartsough EJ (2021) Cell adhesion molecules in plasticity and Metastasis. Mol Cancer Res 19:25–37. 10.1158/1541-7786.MCR-20-0595Epub 2020 Oct 1. PMID: 33004622; PMCID: PMC7785660 33004622 10.1158/1541-7786.MCR-20-0595 PMC7785660

[49] Riley RS, June CH, Langer R, Mitchell MJ (2019) Delivery technologies for Cancer Immunotherapy. Nat Rev Drug Discov 18:175–196. 10.1038/s41573-018-0006-z. PMID: 30622344; PMCID: PMC6410566 30622344 10.1038/s41573-018-0006-z PMC6410566

[50] Sabnis AJ, Bivona TG (2019) Principles of resistance to targeted Cancer Therapy: lessons from Basic and Translational Cancer Biology. Trends Mol Med 25:185–197. 10.1016/j.molmed.2018.12.009Epub 2019 Jan 24. PMID: 30686761; PMCID: PMC6401263 30686761 10.1016/j.molmed.2018.12.009 PMC6401263

[51] Shang S, Hua F, Hu ZW (2017) The Regulation of β-catenin Activity and Function in Cancer: Therapeutic Opportunities. *Oncotarget 8*, 33972–33989, 10.18632/oncotarget.15687. PMID: 28430641; PMCID: PMC5464927 10.18632/oncotarget.15687 PMC5464927 28430641

[52] He Y, Sun MM, Zhang GG, Yang J, Chen KS, Xu WW, Li B (2021) Targeting PI3K/Akt Signal Transduction for Cancer Therapy. Signal Transduct Target Ther 6. 10.1038/s41392-021-00828-5. PMID: 34916492; PMCID: PMC8677728 10.1038/s41392-021-00828-5 PMC8677728 34916492

[53] Yip HYK, Papa A (2021) Signaling pathways in Cancer: therapeutic targets, combinatorial treatments, and New Developments. Cells 10. 10.3390/cells10030659. PMID: 33809714; PMCID: PMC8002322 10.3390/cells10030659 PMC8002322 33809714

[54] Arneth B, Tumor, Microenvironment (2019) *Medicina (Kaunas) 56*, 15, 10.3390/medicina56010015. PMID: 31906017; PMCID: PMC7023392 10.3390/medicina56010015 PMC7023392 31906017

[55] Manduca N, Maccafeo E, De Maria R, Sistigu A, Musella M (2023) 3D Cancer models: one step closer to *in Vitro* Human studies. Front Immunol 14:1175503. 10.3389/fimmu.2023.1175503. PMID: 37114038; PMCID: PMC10126361 37114038 10.3389/fimmu.2023.1175503 PMC10126361

[56] Guo L, Kong D, Liu J, Zhan L, Luo L, Zheng W, Zheng Q, Chen C, Sun S (2023) Breast Cancer heterogeneity and its implication in Personalized Precision Therapy. Exp Hematol Oncol 12(3). 10.1186/s40164-022-00363-1. PMID: 36624542; PMCID: PMC9830930 10.1186/s40164-022-00363-1 PMC9830930 36624542

[57] Ho D, Quake SR, McCabe ERB, Chng WJ, Chow EK, Ding X, Gelb BD, Ginsburg GS, Hassenstab J, Ho CM, Mobley WC, Nolan GP, Rosen ST, Tan P, Yen Y, Zarrinpar A (2020) Enabling technologies for Personalized and Precision Medicine. Trends Biotechnol 38:497–518 Epub 2020 Jan 21. PMID: 31980301; PMCID: PMC7924935 31980301 10.1016/j.tibtech.2019.12.021 PMC7924935

[58] Hu C, Dignam JJ (2019) Biomarker-Driven Oncology Clinical Trials: Key Design Elements, Types, Features, and Practical Considerations. *JCO Precis Oncol 3*, PO.19.00086, 10.1200/PO.19.00086. PMID: 32923854; PMCID: PMC7446374 10.1200/PO.19.00086 PMC7446374 32923854

[59] Arneth B (2019) Tumor Microenvironment. Med (Kaunas) 56:15. 10.3390/medicina56010015. PMID: 31906017; PMCID: PMC7023392 10.3390/medicina56010015 PMC7023392 31906017

[60] Katt ME, Placone AL, Wong AD, Xu ZS, Searson PC (2016) Vitro Tumor models: advantages, disadvantages, variables, and selecting the right platform. Front Bioeng Biotechnol 4:12. 10.3389/fbioe.2016.00012. PMID: 26904541; PMCID: PMC4751256 26904541 10.3389/fbioe.2016.00012 PMC4751256

[61] Jacquemin V, Antoine M, Dom G, Detours V, Maenhaut C, Dumont JE (2022) Dynamic Cancer Cell Heterogeneity: Diagnostic and Therapeutic Implications. *Cancers (Basel)*10.3390/cancers14020280. PMID: 35053446; PMCID: PMC8773841 10.3390/cancers14020280 PMC8773841 35053446

[62] Strickaert A, Saiselet M, Dom G, De Deken X, Dumont JE, Feron O, Sonveaux P, Maenhaut C (2017) Cancer heterogeneity is not compatible with one unique cancer cell metabolic map. Oncogene 36:2637–2642. 10.1038/onc.2016.411Epub 2016 Oct 31. PMID: 27797377; PMCID: PMC5442421 27797377 10.1038/onc.2016.411 PMC5442421

[63] Mandrekar SJ, Sargent DJ (2009) Clinical trial designs for predictive biomarker validation: one size does not fit all. J Biopharm Stat 19:530–542. 10.1080/10543400902802458. PMID: 19384694; PMCID: PMC2931323 19384694 10.1080/10543400902802458 PMC2931323

[64] Fontana F, Marzagalli M, Sommariva M, Gagliano N, Limonta P (2021) In Vitro 3D cultures to Model the Tumor Microenvironment. Cancers (Basel) 13:2970. 10.3390/cancers13122970. PMID: 34199324; PMCID: PMC8231786 34199324 10.3390/cancers13122970 PMC8231786

[65] Fares J, Fares MY, Khachfe HH, Salhab HA, Fares Y (2020) Molecular principles of Metastasis: a hallmark of cancer revisited. Signal Transduct Target Ther 5:28. 10.108/s41392-020-0134-xPMID: 32296047; PMCID: PMC7067809 32296047 10.1038/s41392-020-0134-x PMC7067809

[66] Alday-Parejo B, Stupp R, Rüegg C (2019) Are integrins still practicable targets for anti-cancer therapy? Cancers (Basel) 11:978. 10.3390/cancers11070978. PMID: 31336983; PMCID: PMC6678560 31336983 10.3390/cancers11070978 PMC6678560

[67] Slack RJ, Macdonald SJF, Roper JA, Jenkins RG, Hatley RJD (2022) Emerging therapeutic opportunities for integrin inhibitors. Nat Rev Drug Discov 21:60–78. 10.1038/s41573-021-00284-4Epub 2021 Sep. PMID: 34535788; PMCID: PMC8446727 34535788 10.1038/s41573-021-00284-4 PMC8446727

[68] Ganguly KK, Pal S, Moulik S, Chatterjee A (2013) Integrins and Metastasis. Cell Adh Migr 7:251–261. 10.4161/cam.23840Epub 2013 Apr 5. PMID: 23563505; PMCID: PMC3711990 23563505 10.4161/cam.23840 PMC3711990

[69] Valdembri D, Serini G (2021) The roles of integrins in cancer. Fac Rev 7(10):45. 10.12703/r/10-45. PMID: 34131655; PMCID: PMC8170687 10.12703/r/10-45 PMC8170687 34131655

[70] Mizejewski GJ (1999) Role of integrins in cancer: survey of expression patterns. *Proc Soc Exp Biol Med* ;*222*:124 – 38. 10.1177/153537029922200203. PMID: 10564536 10.1177/153537029922200203 10564536

[71] Hamidi H, Ivaska J (2018) Every step of the way: integrins in cancer progression and metastasis. *Nat Rev Cancer* ;*18*:533–548. 10.1038/s41568-018-0038-z. Erratum in: Nat Rev Cancer. 2019;19(3):179. PMID: 30002479; PMCID: PMC6629548 10.1038/s41568-018-0038-z PMC6629548 30002479

[72] Cruz da Silva E, Dontenwill M, Choulier L, Lehmann M (2019) Role of Integrins in Resistance to Therapies Targeting Growth Factor Receptors in Cancer. *Cancers (Basel)*10.3390/cancers11050692. PMID: 31109009; PMCID: PMC6562376 10.3390/cancers11050692 PMC6562376 31109009

[73] Desgrosellier JS, Cheresh DA (2010) Integrins in cancer: biological implications and therapeutic opportunities. Nat Rev Cancer 10:9–22. 10.1038/nrc2748. PMID: 20029421; PMCID: PMC4383089 20029421 10.1038/nrc2748 PMC4383089

[74] Millard M, Odde S, Neamati N (2011) Integrin targeted therapeutics. *Theranostics*10.7150/thno/v01p0154. PMID: 21547158; PMCID: PMC3086618 10.7150/thno/v01p0154 PMC3086618 21547158

[75] Allison KH, Sledge GW (2014) Heterogeneity and cancer. *Oncology (Williston Park)* ;*28*:772-8. PMID: 25224475 25224475

[76] Hu C, Dignam JJ (2019) Biomarker-driven oncology clinical trials: Key Design elements, types, features, and practical considerations. JCO Precis Oncol 3. PO.19.00086PMID: 32923854; PMCID: PMC7446374 10.1200/PO.19.00086 PMC7446374 32923854

[77] Kwon YW, Jo HS, Bae S, Seo Y, Song P, Song M, Yoon JH (2021) Application of Proteomics in Cancer: recent trends and approaches for biomarkers Discovery. Front Med (Lausanne) 8:747333. 10.3389/fmed.2021.747333. PMID: 34631760; PMCID: PMC8492935 34631760 10.3389/fmed.2021.747333 PMC8492935

[78] Berger MF, Mardis ER (2018) The emerging clinical relevance of genomics in cancer medicine. Nat Rev Clin Oncol 15:353–365. 10.1038/s41571-018-0002-6. PMID: 29599476; PMCID: PMC6658089 29599476 10.1038/s41571-018-0002-6 PMC6658089

[79] Yip HYK, Papa A (2021) Signaling pathways in Cancer: therapeutic targets, combinatorial treatments, and New Developments. Cells 10:659. 10.3390/cells10030659. PMID: 33809714; PMCID: PMC8002322 33809714 10.3390/cells10030659 PMC8002322

[80] Hulsen T, Jamuar SS, Moody AR, Karnes JH, Varga O, Hedensted S, Spreafico R, Hafler DA, McKinney EF (2019) From Big Data to Precision Medicine. Front Med (Lausanne) 6:34. 10.3389/fmed.2019.00034. PMID: 30881956; PMCID: PMC6405506 30881956 10.3389/fmed.2019.00034 PMC6405506

[81] Johnson KB, Wei WQ, Weeraratne D, Frisse ME, Misulis K, Rhee K, Zhao J, Snowdon JL (2021) Precision Medicine, AI, and the future of Personalized Health Care. Clin Transl Sci 14:86–93. 10.1111/cts.12884Epub 2020 Oct 12. PMID: 32961010; PMCID: PMC7877825 32961010 10.1111/cts.12884 PMC7877825

[82] Liu W, Kovacevic Z, Peng Z, Jin R, Wang P, Yue F, Zheng M, Huang ML, Jansson PJ, Richardson V, Kalinowski DS, Lane DJ, Merlot AM, Sahni S, Richardson DR (2015) The molecular effect of Metastasis suppressors on src signaling and tumorigenesis: new therapeutic targets. Oncotarget 6:35522–35541. 10.18632/oncotarget.5849. PMID: 26431493; PMCID: PMC4742122 26431493 10.18632/oncotarget.5849 PMC4742122

[83] Bohr A, Memarzadeh K (2020) The rise of artificial intelligence in healthcare applications. Artif Intell Healthc 25–60. 10.1016/B978-0-12-818438-7.00002-2Epub 2020 Jun 26. PMCID: PMC7325854

[84] Verma M (2012) Personalized medicine and cancer. J Pers Med 2:1–14. 10.3390/jpm2010001. PMID: 25562699; PMCID: PMC4251363 25562699 10.3390/jpm2010001 PMC4251363

[85] Winer A, Adams S, Mignatti P (2018) Matrix metalloproteinase inhibitors in Cancer Therapy: turning past failures into future successes. Mol Cancer Ther 17:1147–1155. 10.1158/1535-7163.MCT-17-0646Epub 2018 May 7. PMID: 29735645; PMCID: PMC5984693 29735645 10.1158/1535-7163.MCT-17-0646 PMC5984693

[86] Lopes-Coelho F, Martins F, Pereira SA, Serpa J (2021) Anti-angiogenic therapy: current challenges and Future perspectives. Int J Mol Sci 22:3765. 10.3390/ijms22073765. PMID: 33916438; PMCID: PMC8038573 33916438 10.3390/ijms22073765 PMC8038573

[87] Khan KH, Yap TA, Yan L, Cunningham D (2013) Targeting the PI3K-AKT-mTOR signaling network in cancer. Chin J Cancer 32:253–265. 10.5732/cjc.013.10057. PMID: 23642907; PMCID: PMC3845556 23642907 10.5732/cjc.013.10057 PMC3845556

